# Machine learning-driven dual-objective optimization of biomass-derived carbon quantum dots toward high conductivity and yield

**DOI:** 10.1039/d6ra02078j

**Published:** 2026-07-06

**Authors:** Heng Jiang, Haoyu Zhang, Ninghui Xu, Yanji Hao, Boyuan Zhang, Jianping Su, Yeqing Li

**Affiliations:** a State Key Laboratory of Heavy Oil Processing Beijing 102249 China jianping.su@cup.edu.cn liyeqingcup@126.com; b Beijing Key Laboratory of Biogas Upgrading Utilization, College of New Energy and Materials, China University of Petroleum (Beijing) Beijing 102249 China; c College of Safety and Ocean Engineering, China University of Petroleum (Beijing) Beijing 102249 China; d School of Advanced Materials, Peking University Shenzhen Graduate School, Peking University Shenzhen 518055 China; e College of Energy Innovation, China University of Petroleum (Beijing) Beijing 102200 China; f Shandong Institute of Petroleum and Chemical Technology, Carbon Neutrality Research Institute Dongying 257061 China

## Abstract

Biomass-derived carbon quantum dots (CQDs) have been reported to effectively improve the efficiency of anaerobic digestion processes. However, simultaneously achieving high CQD yield and high conductivity remains challenging due to the complexity of the process. To address this, a machine learning approach was employed to optimize the preparation of straw-based CQDs. Based on 273 experimentally obtained data points, a predictive model was developed using the AutoGluon framework. Interpretable machine learning analyses were conducted to elucidate the complex relationships between preparation parameters and CQD yield and electrical conductivity. The NSGA-II algorithm was then applied to obtain a set of Pareto-optimal solutions for these dual objectives. Under the high-conductivity scenario, the maximum conductivity of 0.713 mS cm^−1^ was obtained at carbon-to-nitrogen ratio (C/N) = 1 : 2, a hydrothermal temperature (*T*) of 187.7 °C, and a reaction time (*t*) of 323.18 min. Under these parameters, the corresponding optimal CQD yield was 32.02%. Subsequently, we conducted practical anaerobic digestion scenario tests on the optimized CQDs, demonstrating excellent methane productivity, process stability, and acid buffering capacity. These results demonstrate that machine learning can effectively guide the preparation of CQDs with both high conductivity and high yield, and that the prepared CQDs can practically optimize anaerobic digestion application processes.

## Abstract 2

Machine learning-guided optimization enables the synthesis of biomass-derived CQDs with enhanced conductivity and yield, improving anaerobic digestion performance.

## Graphical abstract

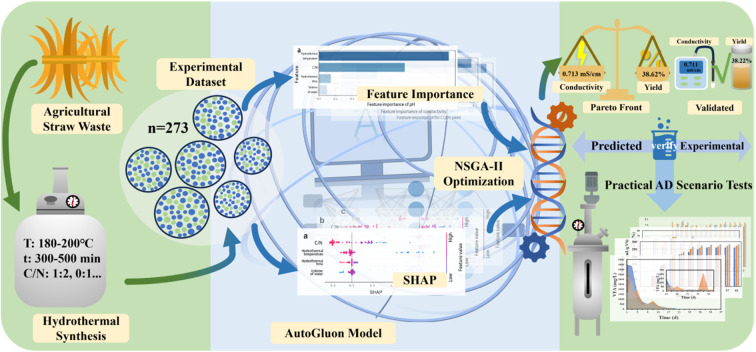

## 1 Introduction

The rapid pace of global industrialization and urbanization has resulted in a substantial increase in the production of organic solid waste.^1^ Anaerobic digestion (AD) is a key technology for converting organic solid waste into resources.^2,3^ It transforms organic solid waste into biomethane and biogenic carbon dioxide, thereby offering significant advantages in achieving carbon emission reduction and energy recovery.^4,5^

One effective strategy to enhance the performance of AD systems is the addition of exogenous conductive materials.^6^ Previous studies have demonstrated that conductive additives can promote direct interspecies electron transfer (DIET), leading to improved methane yield.^7–10^ As a type of quasi-zero-dimensional carbon nanomaterial, carbon quantum dots (CQDs) possess remarkable biocompatibility, high electrical conductivity, and an extensive specific surface area, highlighting their potential for application in AD.^11,12^ Liu *et al.*^13^ first investigated the role of nitrogen-doped CQDs (NCQDs) in AD systems, providing direct evidence for their effectiveness in enhancing process performance.

However, the preparation of biomass-based CQDs as potential AD enhancers faces challenges such as relatively low yield and suboptimal process parameters.^14^ Moreover, the properties of CQDs are influenced by multiple factors, including hydrothermal time, hydrothermal temperature, and the type and concentration of elemental doping.^15–17^ Conventional experimental methods often struggle to fully optimize these complex, interdependent variables, necessitating extensive trial-and-error tests to identify optimal preparation conditions.^18^ This process typically leads to long development cycles and increased experimental costs.^19^ In addition, traditional approaches provide limited insights into the intrinsic relationships between material parameters and final performance.^20^

In recent years, machine learning (ML) has attracted growing attention in materials research.^5,21–23^ ML can learn from data to uncover underlying relationships between parameters and establish efficient mapping models between material states and properties. For example, Wan *et al.*^24^ integrated ML regression models using a genetic algorithm to optimize the electrode structure of a redox flow battery, successfully identifying optimal design parameters. Similarly, Dave *et al.*^25^ employed a Bayesian optimization model to improve the preparation process of non-aqueous lithium-ion battery electrolytes. Leveraging ML to optimize the synthesis conditions of biomass-based CQDs offers a promising approach to reducing experimental effort while providing quantitative insight into the relationships between preparation parameters and CQD properties, thereby facilitating the efficient synthesis of high-performance CQDs.

This study utilizes agricultural straw waste as a precursor to synthesize two types of straw-based CQDs (CQD and NCQD) *via* a one-step hydrothermal method. To address the challenge of simultaneously optimizing CQD yield and electrical conductivity, a machine learning-assisted multi-objective optimization framework was developed. Specifically, the proposed approach integrates interpretable machine learning with genetic algorithms to systematically optimize the preparation parameters. First, the influence of key variables, including hydrothermal temperature, hydrothermal time, water volume, and carbon-to-nitrogen ratio (C/N), on the target properties (yield and electrical conductivity) was investigated. Feature importance and SHAP analyses were employed to quantitatively reveal the contribution and interaction of each parameter. Second, a dual-objective optimization model was established to simultaneously maximize both CQD yield and electrical conductivity using the NSGA-II algorithm. The predicted optimal parameter combinations were further validated through experiments.

Compared with conventional trial-and-error approaches, this study not only improves optimization efficiency and reduces experimental cost, but also provides a data-driven and interpretable strategy for the design and preparation of biomass-derived CQDs. Importantly, the optimized CQDs were further evaluated in anaerobic digestion systems, demonstrating their practical applicability in enhancing methane production and process stability.

## 2 Methods

### 2.1 Data compilation and pre-processing

A dataset comprising 273 data points was generated in this study from laboratory-synthesized straw-based carbon quantum dot (CQD) samples prepared under systematically varied experimental conditions. All experiments were conducted specifically for this work. The samples included both standard straw-based CQDs and nitrogen-doped CQDs (NCQDs) derived from *Chlorella pyrenoidosa*. The dataset comprises four independent variables (feature variables) and three dependent variables (target variables). The independent variables consist of the carbon-to-nitrogen (C/N) ratio, hydrothermal time, hydrothermal temperature, and water volume. The dependent variables include pH, electrical conductivity, and yield. These variables are used to characterize the sample properties and serve as prediction targets, respectively.

Categorical variables were converted into numerical form using ordinal encoding, as summarized in Table 1. Taking the “C/N″ variable as an example, a statistical analysis of its proportional values was conducted to determine their distribution range. Then, based on the magnitude of these proportions, encoding mapping was performed in ascending order. The encoded numerical range is [0, *n* − 1], where *n* represents the number of distinct categories for the variable.

**Table 1:** Coding of C/N textual variables

Column	Category	Encoded value
C/N	0.0 : 1.0	0
C/N	1.0 : 3.0	1
C/N	7.0 : 16.0	2
C/N	5.0 : 11.0	3
C/N	1.0 : 2.0	4
C/N	7.0 : 13.0	5
C/N	3.0 : 5.0	6
C/N	4.0 : 5.0	7
C/N	1.0 : 1.0	8
C/N	13.0 : 7.0	9
C/N	2.0 : 1.0	10
C/N	3.0 : 1.0	11
C/N	10.0 : 3.0	12
C/N	0.3 : 0	13
C/N	1.0 : 0	14
C/N	1.5 : 0	15

Direct numerical representation of the C/N ratio was not adopted due to the presence of extreme or near-zero values in certain experimental conditions, which may lead to instability or undefined ratios. Furthermore, as tree-based machine learning models (*e.g.*, Random Forest, LightGBM, and CatBoost) were employed in this study, the encoding primarily serves to maintain relative ordering rather than impose linear relationships. Therefore, this encoding strategy does not introduce artificial numerical bias and is appropriate for capturing the underlying trends in the data.

*Z*-Score standardization was applied to normalize the entire dataset (excluding C/N) (eqn (2.1)), where a linear transformation was used to scale the original data to zero mean and unit variance.2.1
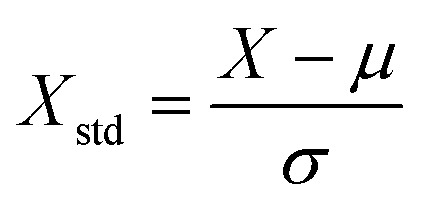
Here, *X* represents the original feature value, *µ* is the feature mean, and *σ* is the feature standard deviation. Through *z*-score standardization, the data are transformed to the same scale, which helps improve model performance and convergence speed.

It should be noted that *z*-score standardization was applied only to continuous variables. The ordinally encoded C/N ratio was not subjected to this transformation, as it represents relative ordering rather than a continuous numerical variable. Applying mean-standard deviation scaling to such ordinal labels does not have a clear statistical or physical interpretation. Moreover, since tree-based machine learning models were employed in this study, the model performance is not sensitive to the scaling of such ordinal variables.

### 2.2 Model training

Following data preprocessing, the original dataset was randomly divided into a testing set and a training set in a 2 : 8 ratio. Training data were applied for model fitting and hyperparameter optimization, while testing data were utilized to evaluate final model performance. The AutoGluon automated ML framework was employed for model construction, within which multiple algorithms were evaluated, including *k*-Nearest Neighbors, Categorical Boosting (CatBoost), Neural Networks, Random Forest, and Light Gradient Boosting Machine (LightGBM).

It should be noted that AutoGluon, under its default settings, incorporates internal cross-validation and bagging strategies during model selection and hyperparameter tuning. Specifically, *k*-fold bagging is automatically applied to enhance model robustness and mitigate potential overfitting, which is particularly important given the relatively limited dataset size.

Separate predictive models were developed for each target variable: pH, electrical conductivity, and CQDs yield. Although pH was not included as an optimization objective in the subsequent multi-objective optimization, it was incorporated as a target variable due to its critical role in reflecting system stability and the chemical environment during CQD preparation. Modeling pH enables a more comprehensive understanding of how synthesis parameters influence process conditions, thereby complementing the analysis of yield and electrical conductivity. In addition, the inclusion of pH contributes to the interpretability of the machine learning model, particularly in SHAP-based analysis and feature importance evaluation, allowing for a more holistic interpretation of the system.

Model performance was evaluated using residual analysis and predicted-versus-observed fitting plots. The prediction residual distribution plot helps assess systematic bias and prediction stability by analyzing the characteristics of residual distribution. The scatter plot of predicted *versus* actual values allows for an intuitive evaluation of prediction accuracy by observing how closely the data points align with the *y* = *x* reference line.

After completing model training and optimization, the most outstanding model was selected for predictions on the testing set. The normalized prediction results were then restored to the original data scale through a denormalization process.

### 2.3 Feature importance analysis and interpretability analysis

Model interpretability was examined using a combination of feature importance analysis and Shapley Additive Explanations (SHAP). This approach was used to quantify the contribution of individual features and to analyze their influence on model predictions.

Feature importance values reflect the degree of contribution of each feature during the model's decision-making process. These values are calculated based on split gain in tree-based models or weight distribution in neural networks. By analyzing the ranking of feature importance, it is possible to identify the most influential features affecting the predictions, thereby providing guidance for feature engineering optimization.

SHAP values, grounded in Shapley value theory from cooperative game theory, assign a specific contribution value to each feature.^26^ This approach precisely quantifies each feature's directional and quantitative contribution to the model's predictions.

### 2.4 Optimal parameter prediction

The Non-dominated Sorting Genetic Algorithm II (NSGA-II) was employed for multi-objective optimization to simultaneously maximize electrical conductivity and CQDs yield. The algorithm was implemented using the Distributed Evolutionary Algorithms in Python (DEAP) evolutionary computation framework. Key algorithm parameters include: population size (*µ*) = 100, offspring size (*λ*) = 100, crossover probability (*p*_x_*b*) = 0.5, mutation probability (*p*_m_*b*) = 0.5, and number of generations (*n*_gen_) = 50.

To address the mixed discrete-continuous nature of the process parameters, an improved genetic operation strategy was designed, which included the following key modifications:

(1) Crossover operation: simulated binary crossover (SBX) was applied to continuous variables, including hydrothermal temperature, hydrothermal time, and water volume, while uniform crossover was used for the discrete variable (C/N). The crossover formula is given by eqn (2.2):

2.2 *X*_new_ = 0.5[(1 + *β*)_*X*_1__ + (1 − *β*)_*X*_2__]

where *β* represents the distribution index.

(2) Mutation operation: polynomial mutation (PM) was applied to continuous variables, whereas a random reset strategy was adopted for discrete variables. The mutation intensity is governed by eqn (2.3):2.3
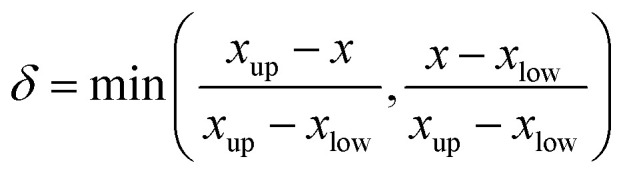
where *x*_up_ and *x*_low_ represent the upper and lower bounds of the parameter, respectively, and the distribution index *η* = 30.

Building upon the optimal model constructed with the AutoGluon framework, a dual-objective optimization function was formulated to simultaneously enhance electrical conductivity and CQD yield. The optimization variables included hydrothermal temperature (*T*, range: 170–190 °C), hydrothermal time (*t*, range: 300–500 min), water volume (*V*, range: 35–50 mL), and 16 predefined carbon-to-nitrogen (C/N) ratio combinations. After 50 generations of evolutionary computation (equivalent to 5100 virtual experiments in total), a Pareto-optimal solution set was obtained.

## 3 Results and discussions

### 3.1 Statistical organization and analysis of raw data

To provide a physicochemical basis for the machine learning analysis, the structural characteristics of the CQDs corresponding to the dataset used in this study were considered based on our previous work under identical synthesis conditions.^27^

Transmission electron microscopy (TEM) analysis revealed that the CQDs possess uniform nanoscale morphology with average particle sizes ranging from 2.6 to 2.9 nm. The lattice spacings were determined to be 0.205–0.217 nm, indicating typical graphitic structures. X-ray photoelectron spectroscopy (XPS) confirmed the presence of abundant surface functional groups, including C–C, C–N, and C=O bonds. Nitrogen doping was verified by the appearance of pyridinic N, pyrrolic N, and graphitic N species, which are known to influence electronic properties. In addition, oxygen-containing functional groups such as C–O–C and C–O–H were identified, which can act as electron transfer mediators. Raman spectroscopy further demonstrated the existence of defect structures and a moderate degree of graphitization, as indicated by the D and G band intensity ratios.

These structural features are directly related to charge transport properties and therefore provide essential support for interpreting the relationship between synthesis parameters and electrical conductivity in the machine learning model.

**Fig. 1:**
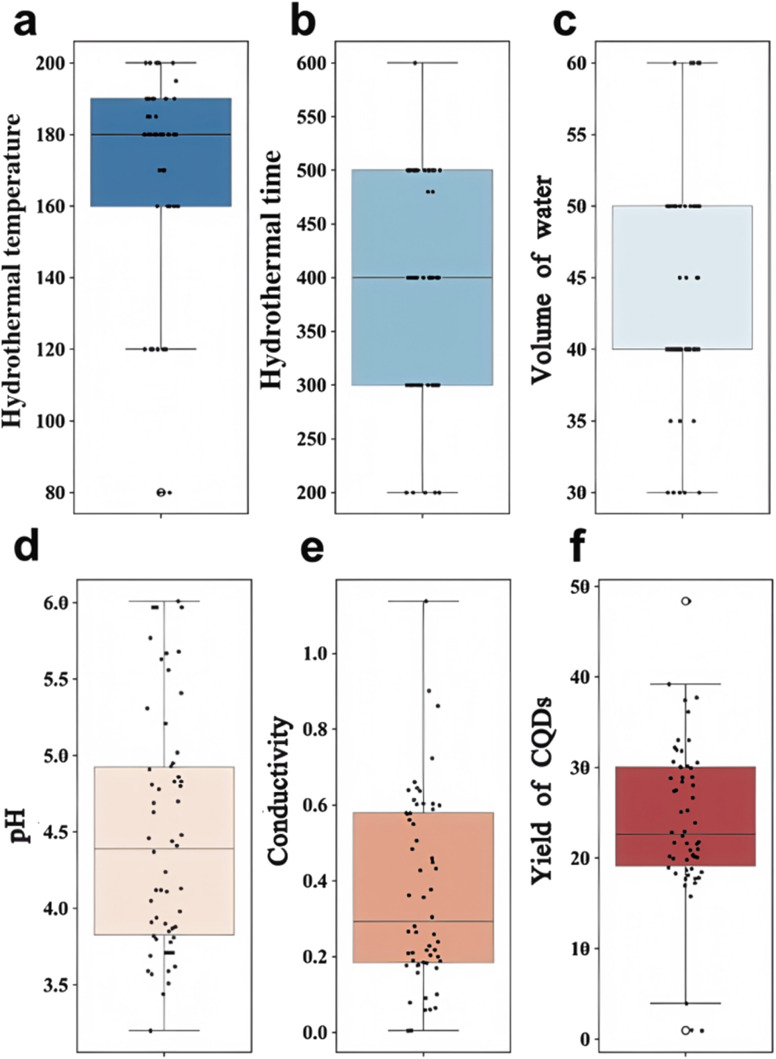
Box plot analysis of the distribution patterns of the key independent variables and dependent variables. Independent variables: (a) hydrothermal temperature; (b) hydrothermal time; (c) value of water. Dependent variables: (d) pH; (e) conductivity; (f) yield of CQDs.

The distributions of key independent and dependent variables are summarized in Fig. 1 using box plots. Hydrothermal temperature was primarily distributed between 120–200 °C, with a median of approximately 170 °C. The short box suggests that the middle 50% of the data is relatively concentrated, but the data exhibits high dispersion and a broad distribution range, reflecting certain fluctuations in temperature control during the CQD preparation process. Fig. 1b indicates that hydrothermal time is primarily distributed between 250 and 600 minutes, with a large box span, suggesting a wide range of control for the hydrothermal time parameter. Water volume exhibited a relatively concentrated distribution, with a median value of about 45 mL (Fig. 1c).

For the dependent variables, Fig. 1d displays a dispersed pH distribution, with data points spanning a broad range (3.5–6.0). Significant pH variation among samples is evident, though the median approaches 4.5, indicating an overall acidic nature of the CQD solutions. Conductivity values are concentrated within 0–1 mS cm^−1^, though data dispersion reflects the ionic concentration characteristics of the CQDs solution. The median conductance is approximately 0.4 mS cm^−1^, with numerous data points distributed on either side of this median, indicating substantial conductivity variation among CQDs samples. Conductivity is closely related to the formation of surface functional groups on CQDs. Analyzing the distribution characteristics of this parameter can further reveal its influence mechanism on CQD performance (Fig. 1e). Fig. 1f shows that the CQD yield distribution ranges from 0 to 50%, with a median of approximately 20%, indicating significant variation in CQD yield.

This box plot system illustrates the distribution characteristics of key parameters during the CQD preparation process. The significant fluctuations in CQD conductivity and yield suggest the potential existence of complex non-linear relationships between independent and dependent variables.

For the pre-processed dataset, Spearman's correlation coefficient was employed to conduct a systematic analysis of feature correlations. A Spearman correlation heatmap was generated, with color gradients mapping correlation values: red regions denote strong positive correlations (coefficient approaching 1), blue regions indicate strong negative correlations (coefficient approaching −1), and light-coloured areas signify no significant correlation (correlation coefficient close to 0).

**Fig. 2:**
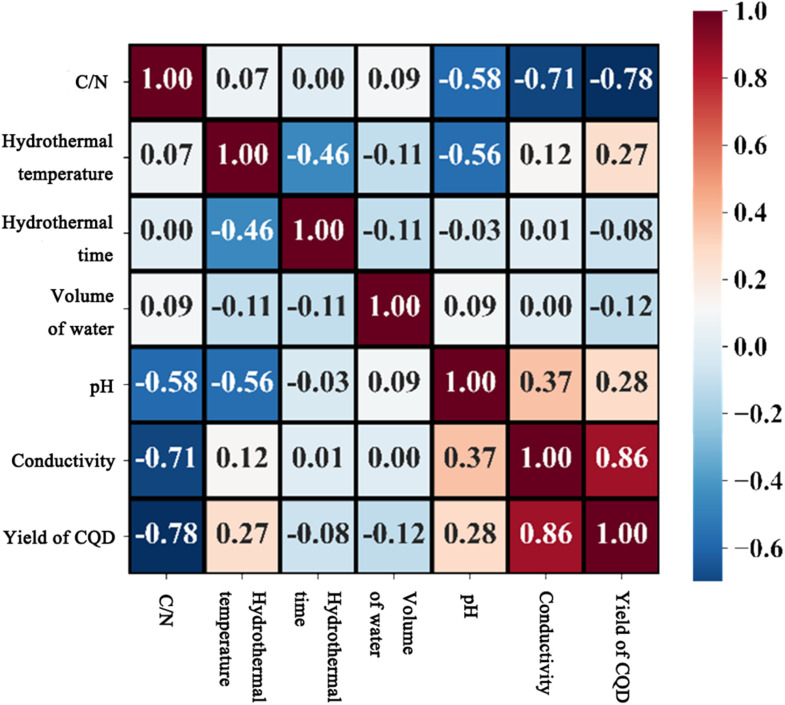
Spearman correlation heatmap.

Spearman correlation heatmaps were used to examine relationships among variables, providing an overview of feature correlations relevant to subsequent analysis. As shown in Fig. 2, the C/N exhibits significant negative correlations with both CQDs yield (*r* = −0.78) and electrical conductivity (*r* = −0.71). This indicates that incorporating a certain amount of *Chlorella* protein during CQDs synthesis from straw carbon sources optimizes the C/N, substantially enhancing both CQDs yield and electrical conductivity.^28^ The correlation coefficient between C/N and pH was −0.58, indicating a certain negative correlation. Hydrothermal temperature and pH (*r* = −0.56) exhibited a negative correlation,^29^ indicating a tendency for pH to decrease with increasing hydrothermal temperature. This may relate to chemical reactions such as hydrolysis and decomposition occurring during the hydrothermal process under elevated temperatures.^30,31^ Hydrothermal temperature showed positive correlations with both conductivity (*r* = 0.12) and CQD yield (*r* = 0.27), suggesting that moderately increasing temperature favors CQD synthesis. Hydrothermal time showed no significant correlation with pH, conductivity, or CQD yield, indicating negligible influence on experimental outcomes. The correlation coefficient between pH and conductivity was 0.37, demonstrating a moderate positive relationship. This suggests that changes in solution acidity affect conductivity, potentially through alterations in ionic concentration or species form within the solution.

### 3.2 Automatic model selection and hyperparameter tuning

For the test set data, following automatic model selection and hyperparameter tuning within the AutoGluon framework, the WeightedEnsemble_L3 model was ultimately selected as the optimal predictive model for modelling all three dependent variables: pH, conductivity, and CQD yield.

**Fig. 3:**
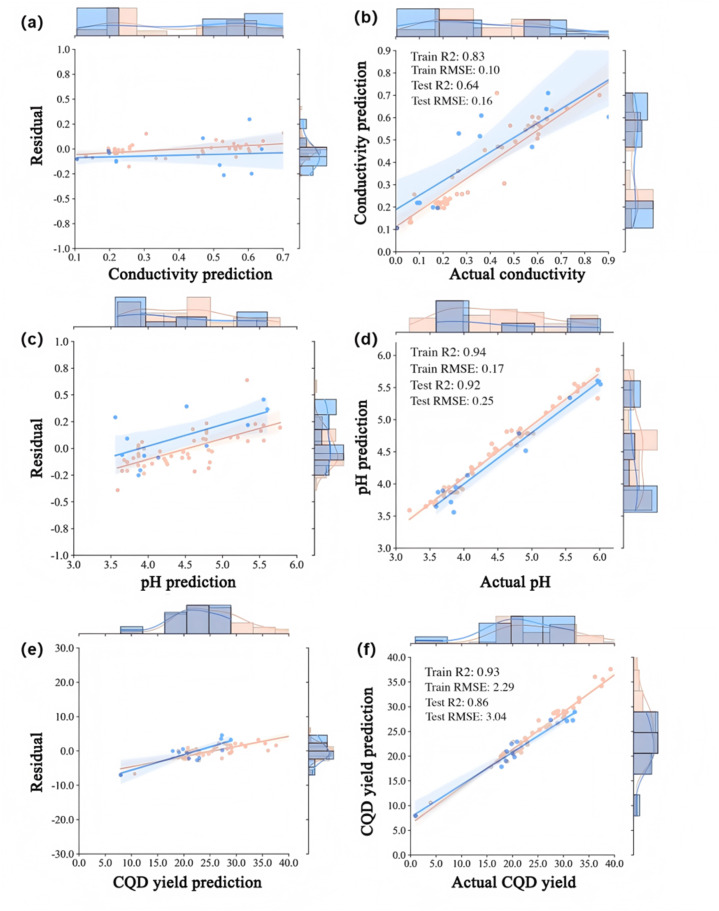
Residual distribution plots on the training (orange) and testing (blue) sets (a, c and e) and fitting scatter plots (b, d and f) (a) conductivity prediction; (b) actual conductivity; (c) pH prediction; (d) actual pH; (e) CQD yield prediction; (f) actual CQD yield.

In the residual plots (Fig. 3a, c and e), orange and blue scatter points represent predictions from the training and test sets respectively. Data points are uniformly distributed near the residual zero line with no discernible systematic deviation, indicating the model exhibits favorable fitting performance and generalization capability.

The fitting performance of the predictive models is illustrated in Fig. 3b, d and f. For electrical conductivity, the model achieved *R*^2^ values of 0.83 and 0.64 for the training and testing sets, respectively, with an RMSE of 0.16. For pH, the training and testing *R*^2^ values were 0.94 and 0.92, respectively, with an RMSE of 0.25. For CQD yield, the model yielded *R*^2^ values of 0.93 for the training set and 0.86 for the testing set, with an RMSE of 3.04. Overall, the similar *R*^2^ values obtained for the training and testing sets, together with relatively low RMSE values, indicate stable model performance and good predictive accuracy for electrical conductivity, pH, and CQD yield.

In addition to the residual plots shown in Fig. 3a, c and e, a quantitative assessment of residual bias was further conducted using the Mean Absolute Error (MAE).

The residuals for the three models are generally centered around zero, suggesting the absence of significant systematic bias. For the conductivity model, the small MAE (0.07) and symmetrically distributed residuals within a narrow range indicate highly stable and accurate predictions. For the pH model, the MAE of 0.17, together with a slight increasing trend in residuals, suggests minor deviation; however, most residuals remain within ±0.2, indicating acceptable predictive performance with limited bias. For the CQD yield model, although the MAE is relatively larger (2.78), the residuals are mainly concentrated within ±3 without obvious skewness, demonstrating good agreement between predicted and experimental values given the larger numerical scale of this target variable.

Overall, both the residual distributions and MAE values confirm that the developed models do not exhibit significant systematic errors and possess reliable predictive capability.

### 3.3 Quantitative analysis of feature importance

**Fig. 4:**
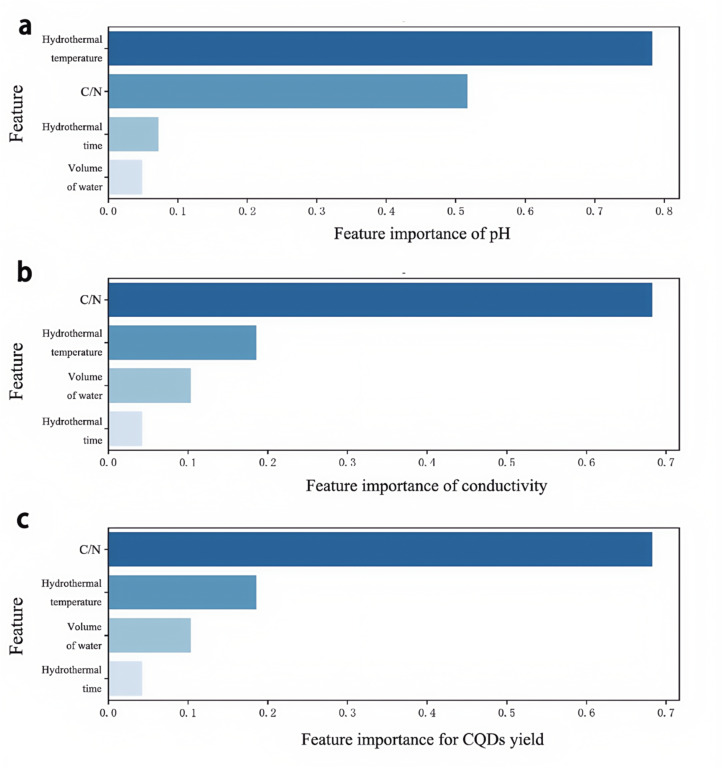
(a) Feature importance of pH; (b) feature importance of electrical conductivity; (c) feature importance for CQDs yield.

Based on the optimal WeightedEnsemble_L3 model, feature importance scores were extracted to evaluate the influence of input variables on the target responses. Fig. 4 illustrates the extent to which different features affect pH, conductivity, and yield during the CQD preparation process.

Fig. 4a presents the feature importance analysis for pH. Hydrothermal temperature exhibits the highest feature importance, with a value approaching 0.8, indicating it is the key factor influencing the final pH of CQDs. C/N follows with an importance of approximately 0.5, suggesting it also significantly affects pH. Hydrothermal time and water volume demonstrate relatively low feature importance, with shorter bar lengths indicating weaker influence on pH.

Fig. 4b presents the feature importance analysis results for conductivity. The C/N dominates with a feature importance value approaching 0.8, confirming its primary role in conductivity. Hydrothermal temperature exhibits an importance of approximately 0.2–0.3, indicating a relatively limited influence on conductivity. Water volume and hydrothermal time demonstrate lower feature importance, suggesting minimal contributions to conductivity from these factors.

Fig. 4c presents the feature importance analysis for CQDs yield. C/N again exhibits the highest feature importance, approaching 0.8, reaffirming its pivotal role in CQDs preparation. Hydrothermal temperature's importance is comparable to that in Fig. 4b, ranging from 0.2 to 0.3, indicating a moderate influence on outcomes. Water volume and hydrothermal time demonstrate lower importance levels, exerting negligible effects on the research results.

Comprehensive analysis reveals that C/N exhibits the highest feature importance for both conductivity and yield, while pH shows the highest feature importance for hydrothermal temperature.^32^ This indicates significant variations in the weighting of feature influences across different target variables. Hydrothermal time and water volume consistently display low feature importance across all three plots, suggesting their effects on outcomes remain relatively stable and limited within the current research framework.

### 3.4 SHAP interpretability analysis

SHAP analysis was employed to investigate the influence mechanisms and interactions of characteristic variables on model prediction outcomes within complex preparation processes.

**Fig. 5:**
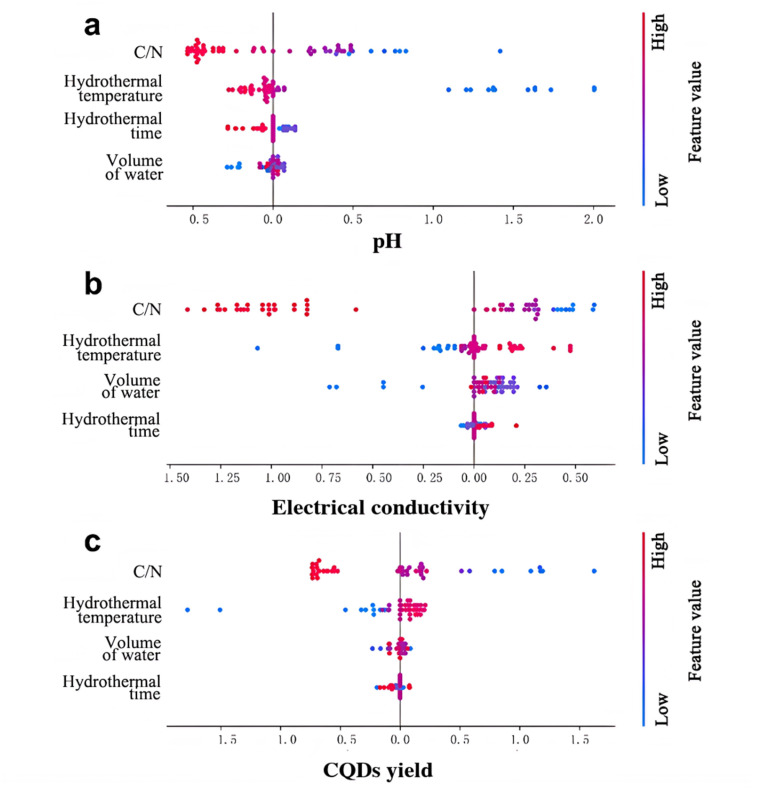
SHAP value analysis chart of C/N, hydrothermal temperature, hydrothermal time, and water volume based on three prediction models (a) pH, (b) electrical conductivity, (c) CQDs yield.

Fig. 5a presents the SHAP-based interpretation of factors affecting the pH of CQDs. Among all preparation parameters, hydrothermal temperature and the C/N ratio are identified as the primary determinants,^33,34^ while hydrothermal time and water volume exhibit negligible influence.

An increase in hydrothermal temperature consistently leads to negative SHAP values, indicating a reduction in pH. This behavior can be attributed to the intensified hydrolysis and decomposition of biomass components into acidic intermediates under elevated thermal conditions.^35,36^ In contrast, the C/N ratio shows a bidirectional and non-linear effect on pH, as evidenced by the coexistence of positive and negative SHAP values. Notably, higher C/N ratios tend to shift pH toward more acidic values, likely due to enhanced incorporation of nitrogen-containing functional groups.^37^

By comparison, hydrothermal time and water volume contribute minimally to pH variation, suggesting that pH regulation during CQD synthesis is predominantly governed by reaction intensity rather than reaction duration or dilution effects.

Fig. 5b displays the results of the SHAP analysis for electrical conductivity. The analysis clearly indicates that the C/N ratio is the dominant factor controlling conductivity, surpassing the influence of all other preparation parameters.

The effect of the C/N ratio on electrical conductivity exhibits a non-linear characteristic, with both positive and negative contributions observed across different value ranges. While positive SHAP values are observed in certain C/N intervals, the overall trend indicates that increasing C/N ratio tends to have a negative impact on electrical conductivity, which is consistent with the Spearman correlation analysis (*r* = −0.71). This behavior suggests that the influence of the C/N ratio on conductivity is complex and cannot be adequately described by a simple monotonic relationship. According to previous characterization results, at lower to moderate C/N ratios, nitrogen doping introduces pyridinic and pyrrolic nitrogen species, which can enhance electron transfer by increasing charge carrier density and modifying the electronic structure of CQDs. This explains the positive contribution of C/N ratio observed in certain regions. However, at higher C/N ratios, excessive nitrogen incorporation may disrupt the conjugated carbon network and reduce the graphitization degree, as indicated by Raman analysis. This structural disruption can hinder electron transport, leading to the overall negative correlation observed between C/N ratio and conductivity.

In contrast, hydrothermal temperature shows a relatively weaker and positive correlation with conductivity, indicating its secondary role in regulating electrical properties.

Hydrothermal time and water volume show SHAP values clustered around zero, indicating that their contributions to conductivity are minimal within the investigated parameter range.

Fig. 5c illustrates the SHAP interpretation of factors influencing CQDs yield. The C/N ratio and hydrothermal temperature emerge as the two most influential variables governing yield performance.

Lower C/N values are generally associated with positive SHAP values, indicating that excessive nitrogen content is unfavorable for CQD formation. This suggests that an appropriate C/N balance is required to promote efficient carbonization and nucleation during hydrothermal synthesis. Consequently, excessively low C/N ratios should be avoided to achieve higher CQD yields.

Additionally, hydrothermal temperature exhibits a pronounced positive contribution to CQDs yield, implying that elevated temperatures promote more complete biomass decomposition and carbonization. This enhanced reaction progression facilitates CQD formation, thereby increasing the overall yield of straw-derived CQDs.

### 3.5 Optimal prediction solution set

The optimal model constructed based on the AutoGluon framework employs the NSGA-II non-dominated sorting genetic algorithm. A dual-objective optimization function was defined to establish an optimization model targeting both maximized electrical conductivity and CQD yield. This yielded a Pareto optimal solution set, with specific results presented in Table 2. Depending on the optimization objective, typical solutions can be categorized into the following two types:

**Table 2:** Pareto optimal solution set

C/N	Hydrothermal temperature (°C)	Reaction time (min)	Water volume (mL)	Conductivity (mS cm^−1^)	CQDs yield (%)
0.0 : 1.0	188.76	424.79	42.89	0.629	38.62
1.0 : 3.0	180.25	408.12	36.79	0.673	35.56
7.0 : 16.0	179.99	312.08	36.83	0.690	33.93
1.0 : 2.0	187.73	323.18	37.45	0.713	32.02

(1) High-conductivity scheme: conductivity reached a maximum of 0.713 mS cm^−1^ at a C/N of 1 : 2, hydrothermal temperature of 187.73 °C, and reaction time of 323.18 min, with a corresponding CQDs yield of 32.02%. This scheme achieves significant optimization of conductive properties by elevating the hydrothermal temperature to near its upper limit (190 °C) and employing a specific C/N combination.

(2) High-yield scheme: at a C/N of 0 : 1, hydrothermal temperature of 188.76 °C, and reaction time of 424.79 min, the CQDs yield peaked at 38.62%, with a corresponding conductivity of 0.629 mS cm^−1^. This scheme indicates that extending the reaction time beyond 400 minutes promotes the complete formation of CQDs, thereby enhancing yield.

Specifically, for CQDs derived from rice straw and *Chlorella vulgaris*, the yield reached a maximum of 35.56% at a C/N of 1 : 3, hydrothermal temperature of 180.25 °C, and reaction time of 408.12 min, with a corresponding conductivity of 0.673 mS cm^−1^.

### 3.6 Experimental validation of predicted results

Based on the optimal preparation parameters obtained from ML multi-objective optimization experiments, validation was conducted through experimental testing. Three parallel experiments were performed for each group, with the final results averaged. The outcomes are presented in Table 3. All experimental results are reported as mean ± standard deviation based on three parallel experiments.

**Table 3:** Experimental validation of Pareto optimal solution prediction results

C/N	Hydrothermal temperature (°C)	Reaction time (min)	Water volume (mL)	Conductivity (mS cm^−1^)	CQDs yield (%)	CQDs yield relative error (%)	Conductivity relative error (%)
0.0 : 1.0	189.00	425.00	43.00	0.609 ± 0.006	38.22 ± 0.04	1.047	3.284
1.0 : 3.0	180.00	408.00	37.00	0.681 ± 0.004	35.26 ± 0.06	0.851	−1.175
7.0 : 16.0	180.00	312.00	37.00	0.680 ± 0.003	34.13 ± 0.08	−0.586	1.471
1.0 : 2.0	188.00	323.00	37.00	0.711 ± 0.007	31.72 ± 0.03	0.946	0.281

The experimental validation results demonstrate that the optimal preparation parameters obtained through the ML multi-objective optimization algorithm exhibit good reliability. As shown in Table 3, when three parallel experiments were conducted using the optimized parameters, the absolute relative error between the obtained straw-based CQD yield and the model prediction values was consistently less than 2%. Similarly, the absolute relative error between the conductivity values and the model predictions was consistently less than 4%, confirming the accuracy of the optimized model.

The CQDs yield and conductivity obtained from experiments conducted using parameters derived from the prediction results both surpassed the original experimental data, validating the model's optimization capability and its enhancement of CQDs preparation performance.

The close agreement between experimental measurements and model predictions demonstrates that the proposed ML-based multi-objective optimization framework is not only statistically accurate but also physically meaningful. The low prediction errors observed for both CQDs yield and electrical conductivity indicate that the model effectively captures the intrinsic relationships between preparation parameters and material properties, rather than relying on overfitting or spurious correlations.

Notably, the optimized preparation conditions simultaneously improved CQDs yield and conductivity beyond the range of the original experimental dataset. This result highlights the capability of the NSGA-II–guided optimization to explore non-intuitive parameter combinations that are difficult to identify through conventional trial-and-error experimentation. Such improvement is particularly significant given the inherent trade-off between yield and functional performance commonly encountered in CQD synthesis.

Moreover, the experimentally validated optimization outcomes are consistent with the mechanistic insights revealed by SHAP analysis, further supporting the robustness of the proposed approach. The dominant influence of C/N ratio and hydrothermal temperature identified in the interpretability analysis is reflected in the optimized parameter set, suggesting that the ML model provides both predictive accuracy and explanatory power. Collectively, these findings demonstrate that ML-driven multi-objective optimization offers a reliable and efficient strategy for guiding the preparation of high-performance biomass-derived CQDs.

### 3.7 Practical application analysis

We systematically investigated the enhancement effect of machine-learning-optimized straw-derived CQDs on anaerobic digestion, focusing on differences in digestion efficiency before and after CQD optimization.

The experimental setup included the blank control group AD, the optimized group AD_ML1_ (1 g L^−1^ CQD) and AD_ML2_ (1 g L^−1^ NCQD). Prior to experiment initiation, high-purity N_2_ was introduced into the fermenter to ensure anaerobic conditions. A constant temperature control system maintained the experimental temperature at 38 ± 1 °C. Biogas produced during fermentation was collected using a 2 L gas bag, and its volume and composition were measured daily. Additionally, 0.25 g of CQDs was replenished into the fermentation system every 15 days.

A 43-day anaerobic digestion experiment was conducted to evaluate the performance of the high-load system by monitoring cumulative methane yield.

**Fig. 6:**
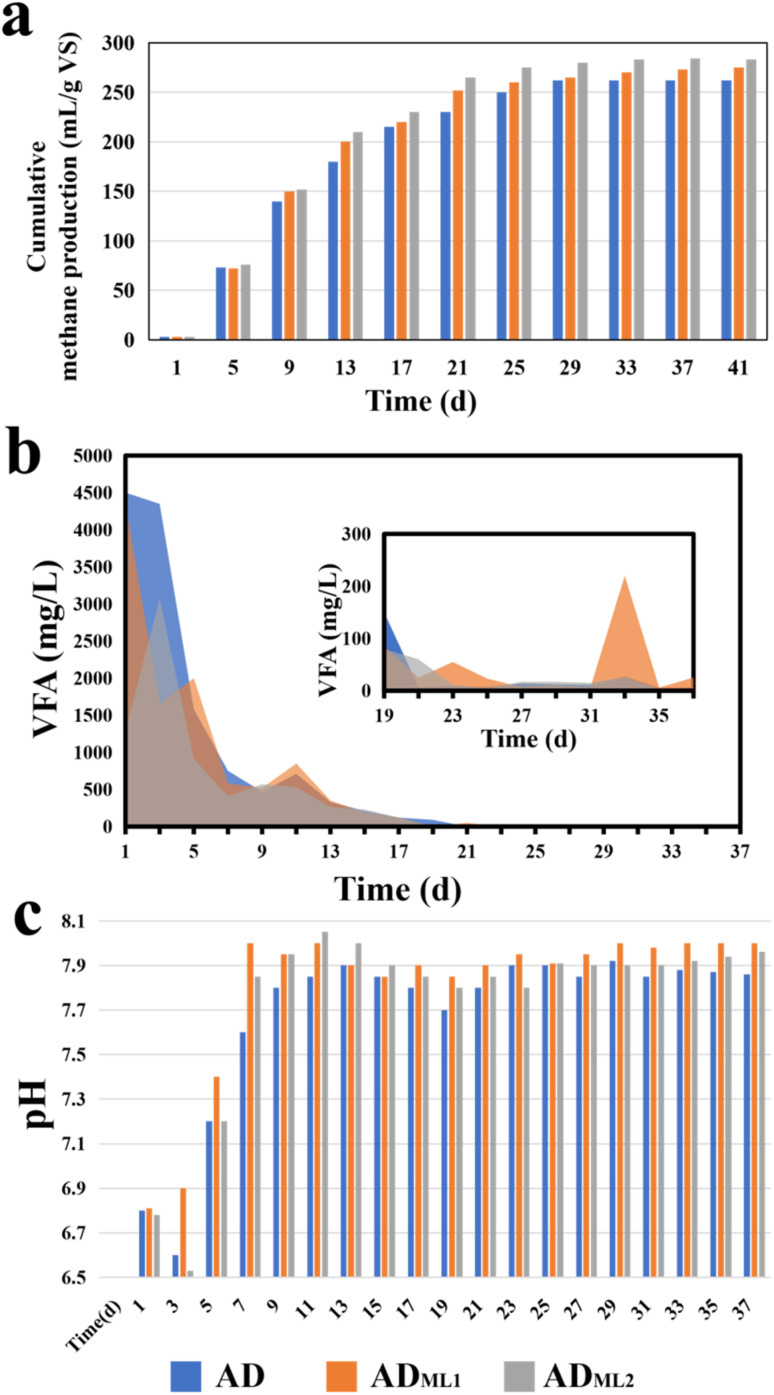
The impact of different CQDs on CH_4_ production under AD and changes in pH and VFA levels. (a) CH_4_ production, (b) VFA, (c) pH.

Results showed that the AD_ML2_ group achieved the highest cumulative methane yield (287.50 mL per g VS), followed by AD_ML1_ (279.69 mL per g VS) (Fig. 6a). Compared with the AD control group (264.07 mL per g VS), cumulative methane yields increased by 8.87% and 5.92%, respectively. These results indicate that machine-learning-optimized CQDs significantly enhance methane production.

During the acidogenic phase of anaerobic digestion, complex organic substrates are degraded by acidogenic bacteria into simpler organic acids. pH and VFA concentration are key factors influencing system performance.

As shown in Fig. 6b and c, pH rapidly decreased during the initial stage due to the conversion of organic matter into VFAs. The pH of both control and experimental groups stabilized at approximately 6.7. By day eight, the pH had gradually returned to a stable state, establishing a dynamic equilibrium. Since most methanogens exhibit optimal pH ranges between 6.8 and 7.5, this indicates that acid inhibition did not occur. The AD_ML2_ group showed rapid pH recovery to around 8 by day 5 (Fig. 6c), whereas recovery was slowest in the control group, indicating slower organic acid consumption and consequently lower methane yield.

During methane production, the cumulative VFA concentration in the control group reached 4507.3 mg L^−1^ on Day 1, and 4367.1 mg L^−1^ on day 3 (Fig. 6b). On day 3, VFA levels in the control group were 1.11 times those of AD_ML1_ and 1.5 times those of AD_ML2_. This accumulation was attributed to slower VFA consumption by methanogens, leading to acid inhibition and reduced microbial activity. In contrast, total VFA content in experimental groups declined significantly after day 5 (Fig. 6b). By day 17, VFA concentrations decreased to relatively low levels (146.7–181.2 mg L^−1^), with AD_ML2_ showing the lowest value (146.7 mg L^−1^).

The practical application results clearly demonstrate that machine-learning-optimized CQDs significantly enhance anaerobic digestion performance under high-load conditions. Compared with the unoptimized CQDs and control group, the optimized CQDs accelerated peak methane production. These improvements indicate that optimization effectively enhanced the functional properties of CQDs, enabling them to better facilitate microbial activity and electron transfer within the anaerobic system.

In addition to methane production enhancement, the optimized CQDs also played a critical role in stabilizing digestion processes. The faster recovery of pH and the rapid reduction of VFA accumulation observed in the optimized CQD groups suggest improved system buffering capacity and more efficient metabolic conversion of intermediate acids. This indicates that optimized CQDs can promote syntrophic interactions between acidogenic bacteria and methanogens, thereby alleviating acid inhibition and maintaining system stability under high organic loading.

Overall, these findings confirm that machine learning serves as an effective strategy for tailoring CQD properties to practical anaerobic digestion applications. By simultaneously improving methane productivity, process stability, and acid regulation capacity, optimized CQDs show strong potential as functional additives for enhancing the efficiency and reliability of large-scale anaerobic digestion systems.

## 4 Conclusions

This study proposes a ML-based framework aimed at optimizing the synthesis process of straw-based carbon quantum dots. The AutoGluon automated ML platform was employed to construct and select predictive models while interpretability analyses were used to elucidate the effects and interactions of key preparation parameters. The results indicate that the C/N ratio exhibits complex effects on pH, while its effects on conductivity and CQD yield follow a more consistent trend. Furthermore, elevated hydrothermal temperatures were found to be beneficial for enhancing CQD yield.

The NSGA-II non-dominated sorting genetic algorithm, implemented *via* the DEAP framework, was applied for multi-objective optimization, providing specific parameter combinations for optimizing the straw-based CQD preparation process: (1) High-conductivity strategy: at a C/N of 1 : 2, hydrothermal temperature of 187.73 °C, and reaction time of 323.18 min, conductivity reached 0.713 mS cm^−1^ with a CQDs yield of 32.02%. The relative yield error was 0.946%, and the relative conductivity error was 0.281%. (2) High-yield strategy: at a C/N of 0 : 1, hydrothermal temperature of 188.76 °C, and reaction time of 424.79 min yielded a conductivity of 0.629 mS cm^−1^, a CQDs yield of 38.62%, a yield relative error of 1.047%, and a conductivity relative error of 3.284%. The carbon quantum dots developed through this guidance demonstrate practical effectiveness in optimizing anaerobic digestion scenarios, validating the application value of machine learning in enhancing performance. Overall, these findings demonstrate that machine learning can effectively guide the optimization of biomass-derived CQD preparation toward simultaneously achieving high yield and high electrical conductivity, highlighting its practical value for process optimization and materials preparation.

## Conflicts of interest

There are no conflicts to declare.

## Abbreviation

AD: Anaerobic digestion
CQDs: Carbon quantum dots
NCQD: Nitrogen-doped carbon quantum dots
DIET: Direct interspecies electron transfer
C/N: Carbon-to-nitrogen ratio
ML: Machine learning
LightGBM: Light gradient boosting machine
DEAP: Distributed evolutionary algorithms in python
CatBoost: Categorical boosting
*k*-NN: *k*-Nearest neighbors
SBX: Simulated binary crossover
SHAP: Shapley additive explanations
PM: Polynomial mutation
NSGA-II: Non-dominated sorting genetic algorithm II
VFA: Volatile fatty acid

## Data Availability

The data supporting the findings of this study are available within the article.
